# 

**Published:** 1852-10

**Authors:** 


### CONTENTS

OF THE

### MEDICAL EXAMINER.

NEW SERIES—NO. XCIV.—OCTOBER, 1852.

### ORIGINAL COMMUNICATIONS.

Experimental Researches applied to Physiology and Pathology. By E. Brown-Séquard, M. D., of Paris.

On the Influence of the Temperature of a Warm-Blooded Animal upon the Duration of its Life when it is Asphyxiated, - - - - - - 617

On the Central Seat of General and of Tactile Sensibility, and on the value of Cries as Manifestations of Pain, - 626

Three Cases of Tape-Worm, with Remarks. By Henry S. Patterson, M. D., Professor of Materia Medica and Therapeutics in Pennsylvania Medical College, - - - - - 629

Notes from my Case-Book. By Wm. H. McKee, M. D., of Raleigh, North Carolina, - - - - - - 634

Dr. S. P. Hullihen's New Method of Filling Teeth over exposed Nerves. Communicated by E. B. Gardette, M. D., - - 641

### BIBLIOGRAPHICAL NOTICES.

Lectures on the Principles and Practice of Surgery. By Bransby B. Cooper, F. R. S., Senior Surgeon to Guy's Hospital, &c., - 643
The Principles and Practice of Surgery. Illustrated by three hundred and sixteen engravings on wood. By William Pirrie, F. R. S. E., Regius Professor of Surgery in the Marischal College and University of Aberdeen, Surgeon to the Royal Infirmary, &c., &c. Edited with Additions by John Neill, M. D., Surgeon to the Pennsylvania Hospital, Demonstrator of Anatomy in the University of Pennsylvania, &c., - 643
Transactions of the Medical Society of the State of Pennsylvania at its Annual Session held in the City of Philadelphia, May, 1852. Published by the Society, - - - - - 652
Sketches of Brazil, including new views on Tropical and European Fever, with remarks on premature decay of the system, incident to Europeans on their return from hot climates. By Robert Dundas, M. D., Physician to the Northern Hospital, Liverpool, &c. &c., - - - - - - 656
A Treatise on the Practice of Medicine. By George B. Wood, M. D., Professor of the Theory and Practice of Medicine in the University of Pennsylvania, &c. &c., - - - - 659
A Treatise on Diseases of the Air Passages: comprising an inquiry into the History, Pathology, Causes and Treatment of those Affections of the Throat called Bronchitis, Chronic Laryngitis, Clergyman's Sore Throat, etc., etc. By Horace Green, A. M., M. D., Professor of the Theory and Practice of Medicine in the New York Medical College, &c. &c., - - - 662
The Physician's Visiting List, Diary, and Book of Engagements, for 1853, - - - - - - - - 662

### EDITORIAL.

Foreign Quackeries and the Drug-Inspection Law, - - - 663
Mineral Springs.—Notice, - - - - - - 666

### MEDICAL NEWS.

Progress of Epidemics, - - - - - - 666
Obituary.—Mr. Herbert Mayo, - - - - - 667

### RECORD OF MEDICAL SCIENCE.

### OBSTETRICS.

Chloroform in Infantile Convulsions and other Spasmodic Diseases. By Professor Simpson, Edinburg, - - - - 667
Discussion in the Academy of Medicine of Paris, on Induction of Premature Labor, - - - - - - 670

### PATHOLOGY AND PRACTICE OF MEDICINE.

Case in which Prune Juice and Orange-Colored Sputa were present without Pneumonia. By J. Wyatt Crane, M. D., Edinburg Licentiate of the Royal College of Physicians, &c., - - 671
Microscopic Examination of a Relaxed Uvula. By T. Inman, M.D., Lecturer on Materia Medica, Therapeutics and Medical Jurisprudence, etc., - - - - - - 674

### MATERIA MEDICA AND THERAPEUTICS.

Remarks on the Effects of Iodine on the Glandular System, and on the Properties of Kousso. By Thomas H. Silvester, M. D., Clapham. (Read at the Anniversary Meeting of the South-Eastern Branch, - - - - - - 675
Lead Poisoning, - - - - - - - 679
On the Galbanum Plant. By F. A. Buhse, - - - - 679

### CHEMISTRY.

Lucifer Match Making and Amorphous Phosphorus, - - 680
Preparation of pure fatty acids for the manufacture of Candles by the distillation of common fats, - - - - 683

